# Draft Genome Sequence of *Chryseobacterium* sp. Strain P1-3, a Keratinolytic Bacterium Isolated from Poultry Waste

**DOI:** 10.1128/genomeA.01237-14

**Published:** 2014-11-26

**Authors:** Gun-Seok Park, Sung-Jun Hong, Chang-Hyun Lee, Abdur Rahim Khan, Ihsan Ullah, Byung Kwon Jung, JungBae Choi, Yunyoung Kwak, Chang-Gi Back, Hee-Young Jung, Jae-Ho Shin

**Affiliations:** School of Applied Biosciences, Kyungpook National University, Daegu, South Korea

## Abstract

*Chryseobacterium* sp. strain P1-3, harboring keratin degrading activity, has recently been isolated from poultry waste. Here, we report the 4.6-Mbp draft genome sequence of the keratinolytic bacterium with a G+C content of 37.0% and 4,087 protein-coding genes.

## GENOME ANNOUNCEMENT

Keratin, the major component of animal hair, nail, feathers, and wool, is the most abundant fibrous protein in the epithelial cells of vertebrates (1). The insoluble protein is tightly packed in an α-helix structure (α-keratin) or β-sheet structure (β-keratin) into a supercoiled polypeptide chain with cysteine bridges (2). Keratin shows resistance to proteolysis due not only to the disulfide bonds, but also to the structurally limited interior space with hydrophobic interactions between nonpolar residues (3). The proteolytic resistance of keratin has been considered to be a critical factor disturbing the process of keratin waste treatment (4). To date, some keratinolytic microorganisms producing various proteases have been reported, such as *Bacillus* (5), fungi (6), thermophilic bacteria (7), and even *Chryseobacterium* (8). The keratinolytic proteases from these organisms may have important uses for the biodegradation of keratin-containing waste from industry (9).

The genus *Chryseobacterium*, belonging to a member of family *Flavobacteriaceae*, is a non-spore-forming, nonmotile, rod-shaped Gram-negative bacterium. *Chryseobacterium* species have been known to possess strong proteolytic activity with typical morphological characteristics, such as translucent circular and yellow pigmented colonies (10). A strong keratin degrader, strain *Chryseobacterium* sp. P1-3 was isolated from a poultry waste dumping ground in Iksan, Jeonbuk, Republic of Korea (S. J. Hong, unpublished data). The genomic DNA of the strain P1-3 was sequenced to enlarge the enzymatic hydrolysis efficacy for keratin waste treatment.

The genome was sequenced using an Ion Torrent personal genome machine (PGM) sequencer with a 316 v2 chip (11). The sequence reads were assembled using Mimicking Intelligent Read Assembly (MIRA) 4.0 (12). The draft genome is composed of 45 contigs (>728 bp). The genome size is 4,628,764 bp at 78.0-fold coverage with a G+C content of 37.0%. The assembled contigs were annotated using the Prokaryotic Genome Annotation Pipeline (PGAP) version 2.6 software on NCBI (http://www.ncbi.nlm.nih.gov/genome/annotation_prok/) and the Rapid Annotation using Subsystem Technology (RAST) server (13). The genome contains a total of 4,087 genes, consisting of 3,119 protein-coding sequences (CDSs), 882 pseudo genes, 20 rRNA (5S, 16S, 23S) genes, 65 tRNA genes, and 1 ncRNA gene. Predicted genes were functionally assigned via 311 RAST subsystems. In regard to the keratinolytic activity, a total of 32 open reading frames are identified as proteases, including metalloproteases and serine proteases.

The genome sequence of *Chryseobacterium* sp. strain P1-3 may contribute to a better understanding of keratin degradation and also provide potential applications for the treatment of keratin wastes.

### Nucleotide sequence accession number.

The draft genome sequence of strain *Chryseobacterium* sp. P1-3 has been deposited at DDBJ/EMBL/GenBank under the accession no. JPEQ00000000.
